# Some Peculiarities of Using the Extended Finite Element Method in Modelling the Damage Behaviour of Fibre-Reinforced Composites

**DOI:** 10.3390/ma18081787

**Published:** 2025-04-14

**Authors:** Vladislav Kozák, Jiří Vala

**Affiliations:** Institute of Mathematics and Descriptive Geometry, Faculty of Civil Engineering, Brno University of Technology, 613 00 Brno, Czech Republic; vladislav.kozak@vut.cz

**Keywords:** fibre composites, crack initiation and development, computational modelling, extended finite element method (XFEM), 62.20.mt, 46.50.+a, 02.70.Dh, 02.70.Bf

## Abstract

The present study utilises the extended finite element method (XFEM) to model fibre-reinforced composites, with a focus on crack initiation and propagation. Silicon nitride-based ceramics were selected as a model material; they represent a broad class of short fibre ceramics and have received a lot of attention in recent decades. Some peculiarities when using the XFEM, including its selected modifications, are discussed in response to applied external stresses, mainly in the viscoelastic range. Promising approaches are recommended, which lead to a more accurate description of these materials under operating conditions, focusing on the correct calculation of the macroscopic stress ahead of the propagating crack front. The authors draw on years of experience with the material and investigate the possible improvements and modifications to the XFEM.

## 1. Introduction

The concept of fibre-reinforced composites covers a wide class of modern constructive materials, well-known for their considerable strength and stiffness, frequently exploited in both building and mechanical engineering, namely, in various types of engineering structures, in the automotive, maritime, and aerospace industries, and in sport goods production. They consist of fibres from various materials, such as steel, glass, carbon, aramid, etc., including natural (plant, animal, or mineral) source fibres and fibres coming from waste and recycled materials. The mechanical, thermal, etc., properties of such composites depend on those of the following: (i) their fibres, considered as some discontinuous or dispersed phase; (ii) their matrix as a certain continuous phase, which is usually a cementitious one in most building composites, or a ceramic one where ceramic fibres are embedded in a ceramic matrix again; (iii) a set of interphase regions, usually addressed as the interface.

The behaviour of material samples, the structural parts of whole structures under mechanical, thermal, etc., loads, is conditioned by the relevant active physical (and maybe also chemical, biological, and other) processes. The fundamental thermomechanics conservation principles of mass (linear and angular), momentum, and energy, as defined in [1], supplied by some constitutive relations, with material parameters for engineering calculations obtained from some sufficiently simple and well-arranged experiments, cf. [2], is the foundation of this paper. A crucial characteristic for the durability of structures based on fibre-reinforced composites, needed in the design phase of any fibre-reinforced composites already, is some reasonable quantitative estimate of the danger of (1) the initiation of microscopic fractures and (2) the development of macroscopic cracks, leading to total destruction. The prevailing mechanisms of (1) and (2) depend on the choice of (i), (ii), and (iii). Following the classification of [3], (a) a ductile fracture involves remarkable plastic deformation at the crack tip, whereas (b) a brittle fracture initiates with little or no plastic deformation there, which can rapidly result in the passage from (1) to (2). Moreover, (c) a quasi-brittle fracture can be characterized by two distinguishable stages, (1) and (2), as follows: micro-cracks develop, grow, and join together, resulting in macro-cracks. Examples of (a) are steel-based and nickel-based superalloys; examples of (b) are fibre-polymer composites or various ceramic materials, as well as rock massifs; and examples of (c) are most cementitious composites, such as steel fibre-reinforced concrete.

The prediction of the development of deformation and stress fields in test specimens and, subsequently, in real structures is based on the above-mentioned physical formulations, which are simplified for the design of a computational model based on practical experience. This leads to the numerical solution of partial differential equations with the appropriate initial and boundary conditions. Their solutions, except for some artificial benchmark cases, are rarely available in analytical or semi-analytical form, namely, as sums of the appropriate infinite series. Therefore, some weak or variational formulations of such equations (or their systems) are needed. Following [4], Chapter 8, a typical computational approach then couples the method of discretisation in time (of Rothe sequences), or its certain simplification for a quasi-static case, with some modification of the finite element method (FEM), as some variant or improvement of the extended finite element method (XFEM), formulated by [5]. The rapid development of such methods, in recent years, is documented in [6], where the phantom node version of the XFEM in [7] is confronted with that of [8], working with a user-defined crack front zone, open to 3D dynamical simulations. For the relations of such approaches to cohesive zone modelling, useful for fibre-reinforced composites, cf. [9]. Alternatively, one can start with the Fourier multiplicative decomposition of the unknown solution (method of lines) and come to the same or similar algebraic systems, generated by FEM/XFEM again. For the exploitation of fundamental solutions of particular elliptic problems, see [10]. For modern insights into the theory and practice of a rather wide class of meshless methods cf. [11]. However, this apparently simple technique introduces serious difficulties both in the formal mathematical theory of the existence, uniqueness, or so-called weak solutions, including the convergence of sequences of approximate solutions in finite-dimensional spaces, and in the design of robust and efficient computational algorithms with reliable results for engineering applications, which can often be considered as conflicting requirements. Consequently, regardless of the 25 years of development of such modelling and simulation mathematical and computational tools, including software implementations, a lot of open problems and challenges for deeper analysis persist in this research area.

All considerations in this article will reduce such a wide research scope to some peculiarities with using the XFEM, including its selected modifications to the above sketched analysis, with emphasis on the remarkable research progress in the last several years, which is not covered by clearly structured review articles. After this introduction, Section 1, Section 2 summarises the necessary physical preliminaries for the above sketched computational techniques. Section 3 is devoted to the overview of XFEM-based approaches. In the context of this, Section 4 presents some illustrative examples from the research practice of the authors. Section 5 then suggests some recent XFEM-motivated methods and their relationships to the previous methods. Section 6 summarises all the results, including the highlighted directions for further research.

## 2. Physical Preliminaries

Silicon nitride-based ceramics, which include a wide class of short fibre ceramics and have garnered significant interest in recent decades, have been chosen as a model material because of their exceptional room and high-temperature features. They demonstrate grain bridging, which increases toughness and strength. The prediction of crack development through interface elements has been investigated using a quantitative damage model and the fracture mechanics strategy with a cohesive zone model utilising the XFEM. For contemporary bearing applications, ceramics like silicon nitride Si_3_N_4_ have been accepted as the best candidate. However, we should consider the severe working conditions that must be tolerated, such as high temperatures, corrosive environments, and rolling, in locations where, throughout service, the elements are exposed to significant cyclic contact stresses [12].

It is commonly known that appropriately adjusting the microstructure depends on the practical application and operating conditions, which can significantly improve the characteristics of ceramics. More specifically, Ref. [13] showed how the fracture toughness of silicon nitride could be changed by modifying the grain shape, among other things. Similarly, the influence of boundary phase manipulation and the effect of grain bridging on the strength and toughness is shown in [14]. Enhancing the fracture resistance of ceramics requires an understanding of the nature of crack formation in ceramic materials and how they relate to the behaviour of the resistance curve (*R* curve) [15]. Recently, however, it has been shown that these mechanisms fall short in explaining the significantly increasing fracture resistance curves (*R* curves) observed for some materials [16]. Innovative approaches for determining bridging stresses [17] have also been proposed.

**Figure 1:**
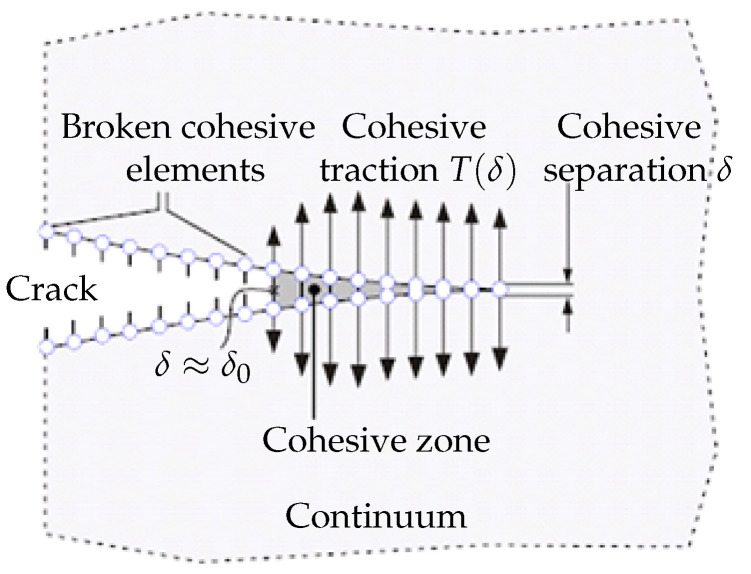
Crack tip growth based on the cohesive zone model.

The special element implementation responds to damage (cohesive elements) or the XFEM with traction separation law for crack growth modelling, introducing the so-called “damage mechanics”. Both principles are similar; the origin can be found in the contact elements and in the vanishing elements, and the new surface is created. A realistic description of the material characterising the behaviour of a given composite is realised by means of a traction separation law, describing the local damage predicted by the total strain energy. New special models published in recent years can then be found in the literature for laminates, composites, long-fibre composites, etc. The physical interpretation of the cohesive zone is still a subject of discussion, even though cohesive zone modelling has been used for more than 20 years. Due to its almost negligible thickness and size, this zone may not be completely consistent with traditional fracture mechanics procedures. Characteristics of the actual cohesive zones $\left(T,\delta_{0},J_{0}\right)$, as sketched by Figure 1, can be derived by the strain and stress analysis in narrow bands. Since the cohesive model is a phenomenological model, there is no evidence of which form is to be taken for the cohesive law. Thus, the cohesive law has to be assumed independently of the specific material, as a model of the separation process. Most authors take their own formulation for the dependence of the traction on the separation. With unloading constantly going back to the origin, the T-δ response maintains an irreversible trajectory.

One of the best ways to guarantee adherence to the experiment and prediction is to simulate damage using composites based on an understanding of the key micromechanisms. These fundamental micromechanisms then characterise the damage when the fracture spreads in the composite, in a direction perpendicular to the reinforced fibre or grain, as follows: matrix cracking, delamination of interface fibres (grains) and matrix, fibre (grain) cracking, and pull out. Predicting the behaviour of the contact between the matrix and the fibre (grain) is the crucial issue. The determination of fracture toughness, fracture strength, and overall fracture behaviour is greatly influenced by this interaction.

From a micromechanical perspective, each component has a unique microstructure that results from the representative volume element (RVE) method. The cohesive element describes and determines material separation and damage. In this way, we divide material behaviour into two areas that are very different from one another. Both the cohesive model and the fracture mechanism, which are schematically depicted in Figure 1, illustrate how cracks propagate through an element. When simulating fracture growth and fragmentation in metals, polymers, and ceramics, cohesive models are frequently employed.

In order to perform a general constitutive modelling of materials, whose fracture can be explained by cohesive cracking, the following three key components must be defined: (1) The stress–strain behaviour of the material as characterised by traditional constitutive modelling when cohesive cracking is absent. (2) The initiation criterion, which establishes the orientation of the newly created cohesive fracture and the circumstances under which a crack forms. (3) The cohesive crack’s evolution rule, which links the relative displacement between the crack lips to the stresses transmitted between its faces.

**Figure 2:**
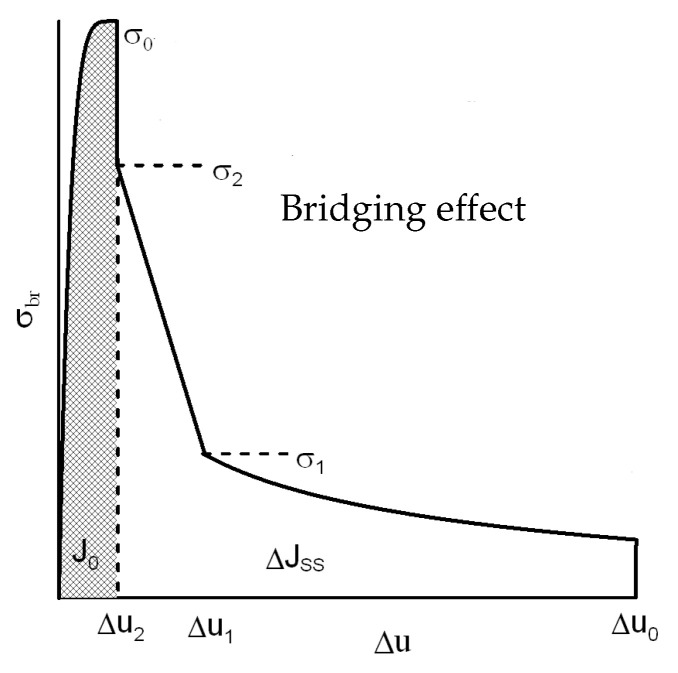
Shape of the traction separation law.

All cohesive laws share a few common characteristics as follows: (a) they consist of two material parameters $\delta_{0}$ and $T_{0}$, and (b) stress falls to zero with material damage, $T(\delta >\delta_{0})=0$ for normal and tangential separation (this criterion is not precisely reached for all cohesive laws). The energy spent by the cohesive law is defined by the area below the traction–separation curve, whether in a tangential or normal direction. Figure 2 shows a schematic diagram for the composite. From a numerical point of view, it is visible that this shape of the traction–separation law is the source of instabilities, and the numerical solution is going to diverge. A leading edge up to the peak stress reflects the Dirac function. Smoothing techniques away from the singularity are needed for $\Delta_{u}=0$, respectively, and the strength $J_{0}$ is introduced. Crack creation and extension can be forecast with fracture mechanics if a correlation between the bridging stress $\sigma_{br}$ and fracture energy is identified.

At every location within the bridging zone, the bridging law, expressed as σ=σ(δ), remains identical. Grain damage starts immediately in the case of shock loading; therefore, it is necessary to suppose that there is a defining opening $\delta_{0}$ that establishes the point at which the bridging effect disappears. Despite the fracture resistance curve (*R* curve or *J*-δ curve), the bridging law, such as the material characteristic, is accepted. Using the *J* integral on the crack surface and the crack area to partition the fracture energy results in

$$J=\int_{0}^{\delta^{*}}\sigma \left(\delta \right)\;d\delta +J_{TIP}$$

where the $J_{TIP}$ denotes the *J* integral assessed around the crack tip (while cracking is equal to the fracture energy of the tip, $J_{0}$); see Refs. [18,19] for significantly more detail on similar integral classes. The bridging zone subsequently releases the entire energy, and the maximum opening of the bridging zone at the notch root is $\delta^{*}$. The bridging law is then determined as

$$\sigma \left(\delta^{*}\right)=\partial J_{R}\left(\delta^{*}\right)/\partial \delta^{*}$$

where the value of the *J* integral during the fracture growth is denoted by $J_{R}$. The bridging stress is absent from the fracture at first, and the initiation occurs when $J_{R}=J_{TIP}=J_{0}$. We can find specific versions of the bridging law in Ref. [20]. The following formula can be used to characterise the shape of the bridging law, as follows: the steady-state value of fracture energy is determined at the bridging zone’s end

$$J_{R}\left(\delta^{*}\right)=J_{0}+\Delta J_{SS}{\left(\delta^{*}/\delta_{0}\right)}^{1/2}\;.$$

**Figure 3:**
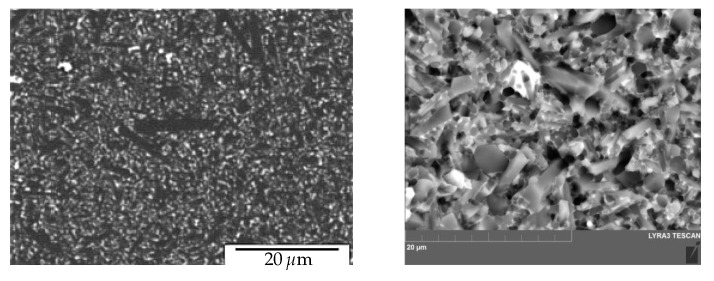
Fracture and original surfaces of the typical silicon nitride composite; optical microscope snapshots.

**Figure 4:**
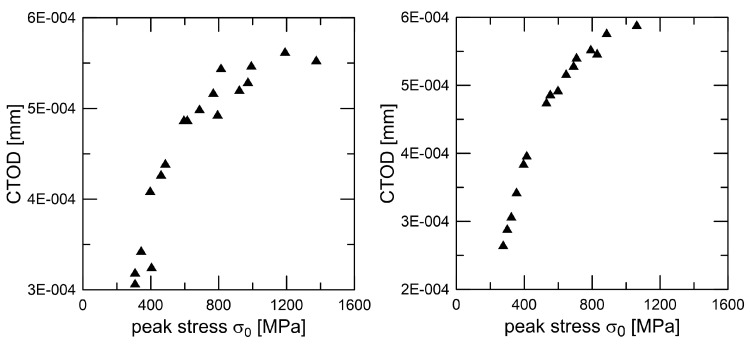
Peak stress determination for two selected modification of the silicon nitride composite.

**Figure 5:**
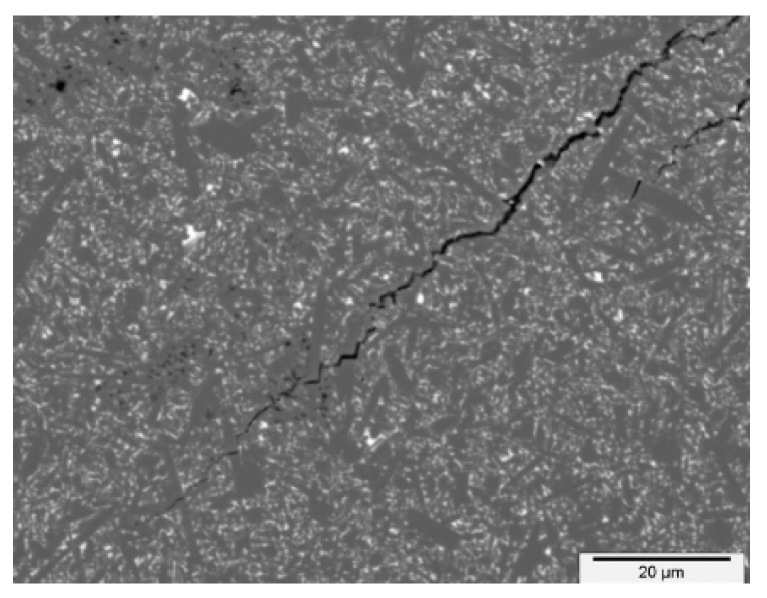
Detailed view of the crack part, true length is 45 µm, measured via the optical microscopy.

The cohesive rules can be implemented in a variety of ways within the commercial standard FEM package. The authors’ extensive knowledge of the ABAQUS system makes it possible to integrate material alterations to this package in a highly effective way, which ultimately affects the structure of the traction separation law. The function

$$\sigma_{br}\left(\delta \right)=\frac{\Delta J_{SS}}{2\delta_{0}}{\left(\delta /\delta_{0}\right)}^{-1/2}$$

is described in the literature as being quite practical for use on composites. Additional details are available in [16]; in particular, the model application benefited from the bridging stress prediction. An impulse stimulation approach has been used on the set of samples in order to measure the elastic modulus. Two directions, perpendicular to the compressing direction and in the pressing direction, were examined. Because $E_{A}$ = 293.07 GPa and $E_{B}$ = 293.83 GPa, the degree of anisotropy in elastic modulus values is quite low (around 1 GPa). Poisson’s ratio has been determined to be 0.283. The typical fracture surface can found in Figure 3, and the procedure for peak stress determination (for traction separation law) for two similar materials Si_3_N_4_ can found in Figure 4. A detailed view of the short crack is provided in Figure 5. For good prediction, it is important to find the real value of peak stress $\sigma_{0}$. The exact determination of the peak stress for the traction–separation law is absolutely necessary in the case of lower values of fracture toughness.

**Figure 6:**
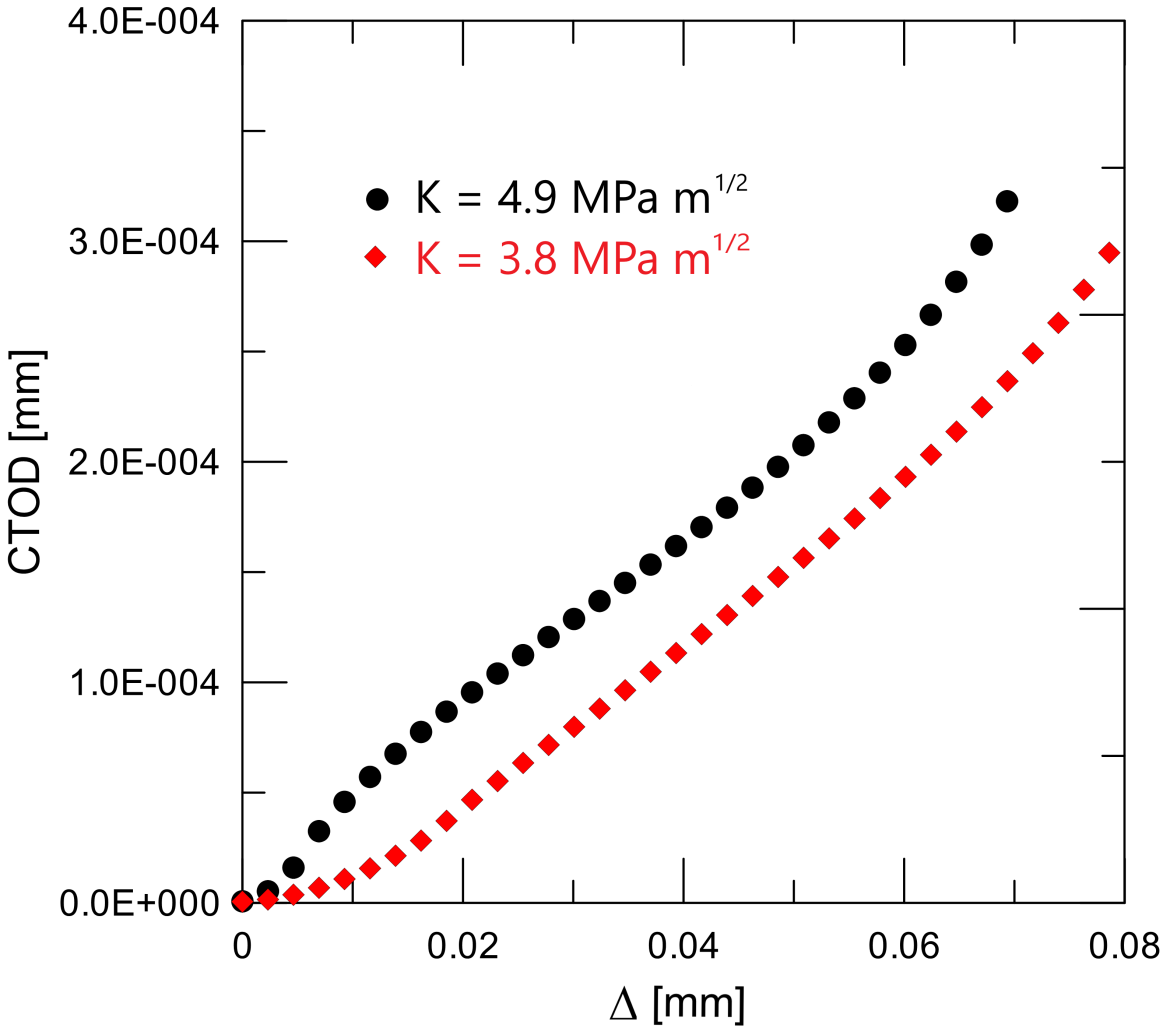
Crack tip opening displacement (CTOD): experimental data versus crack length.

**Figure 7:**
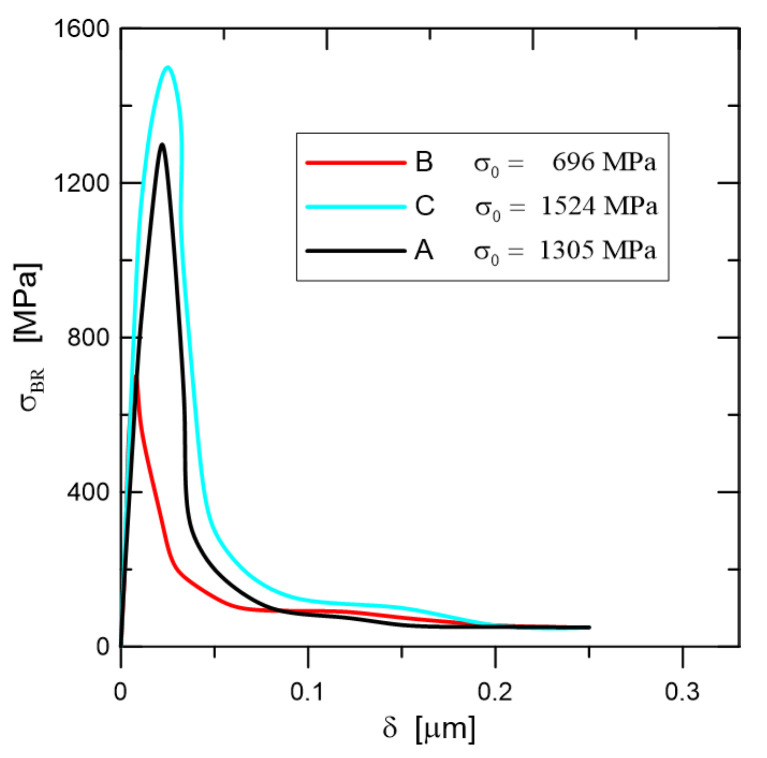
Bridging law after calibration procedure for materials A, B, C.

Data presented in Table 1 were obtained via the above-mentioned procedures for fracture toughness determination and are shown in Figure 6; after calibration, we can obtain Figure 7. Modified new approaches are well explained in [21,22].

Some views from electronic, atomistic, microscopic, mesoscopic, micromechanics, mesomechanics, etc., scales, as discussed in [23], Chapters 5, 6, and 7, are provided in great detail, namely, for a certain supercomputer-supported scale bridging approach for crack propagation in a brittle specimen in Part 7.10. Although such an expensive approach is impossible for standard XFEM-based computations, some recent approaches coupling mesoscopic and macroscopic scales for various fibre-reinforced composite structures, relying on certain combinations of discrete element and finite element techniques, are presented in [24,25].

## 3. Mathematical Modelling and Computational Implementation

From such numerical methods, ref. [26] has garnered the most attention for the new numerical method based on FEM, which led to the development of a method later referred as XFFEM. It also allows for the elegant alleviation of remeshing by representing discontinuities and singularities in the function space, as was mentioned previously.

In the meantime, the local enrichment occurs according to a priori skills, producing a highly precise solution. Such an enrichment function design, especially on the crack front, is rather delicate; for more details on the development of the related numerical methodologies, including the practical computational formulae, cf. [27,28]. Because of all these exceptional qualities, the XFEM has become well known in the industrial community and has been integrated into a number of commercial software programs (such as ABAQUS 2024, ANSYS 2024 R2, etc.).

Due to the relevance of the evaluation of damage risk in several engineering and applied research areas, the development of modified finite element and similar techniques for such evaluations has its own history, whose origin dates back to the end of the twentieth century. For the remarkable progress in the last 30 years, one can compare surveys from the pioneering period [26,29] with those from the last several years [30,31], regardless of their limitations to special classes of composites, especially for fibre-reinforced composites [32,33], enriched by the exploitation of machine learning [34].

From a historical point of view, one can distinguish, in addition to the standard FEM techniques, criticised for their difficult applications to cracked domains, several slightly different methods, such as the partition of unity FEM (PUFEM) in [35], generalised FEM (GFEM) in [36], classical (extrinsic) extended FEM (XFEM) in [37], and intrinsic version of FEM (without increasing DOFs (degrees of freedom)) in [38]. For a comparison of these approaches, see [39], and for the classification of their various modifications and for some concurrent approaches presented in the last decade, cf. [40].

The idea of crack initiation, closing, opening, and development, based on a certain class of traction separation laws, motivated by [41], microstructurally, is elaborated in [20,42]. Nevertheless, these approaches rarely deal with fibre-reinforced composites, where crack initiation and development is possible (i) at (a priori known) matrix - particle interfaces, (ii) inside a matrix, and (iii) inside some fibres. The first step for the detailed analysis of the damage in fibre-reinforced composites is the proper evaluation of strains end stresses in all their components. In Refs. [43,44], this relies on certain viscoelastic material computational models. The second step is the proper incorporation of (i), (ii), and (iii), using the XFEM, together with some version of the method of discretisation in time, or its certain quasi-static simplification. For several examples of such incorporation attempts, see [45,46,47,48].

## 4. Illustrative Examples

**Figure 8:**
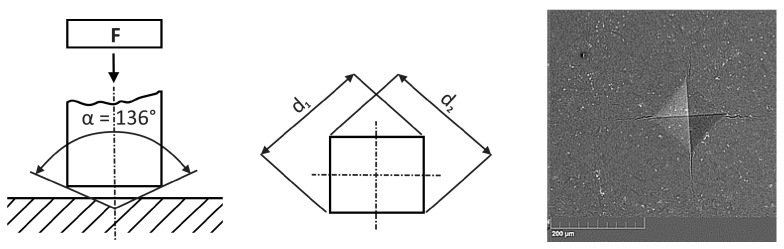
Indentation technique: schematic diagrams and indenter impression.

The indentation technique was used to examine the fracture behaviour of small cracks, as is shown in the left-hand side of Figure 8, and this test was modelled using the FEM and XFEM techniques. Indentation tests are the basis for characterising the mechanical properties of both the surface layer itself and the entire system and the surface of the base material. The principle consists of penetrating the test specimen, the indenter, into the material under examination and subsequently determining the degree of deformation of this material. In this case, a diamond tetrahedral pyramid with an apex angle of 136° is used as the indenter (hardness test). By applying different indentation loads, this technology made it possible to observe the behaviour of cracks, both while they are starting and when they are spreading. A typical example of an imprint and the corresponding cracks is shown in Figure 8 (right figure).

**Figure 9:**
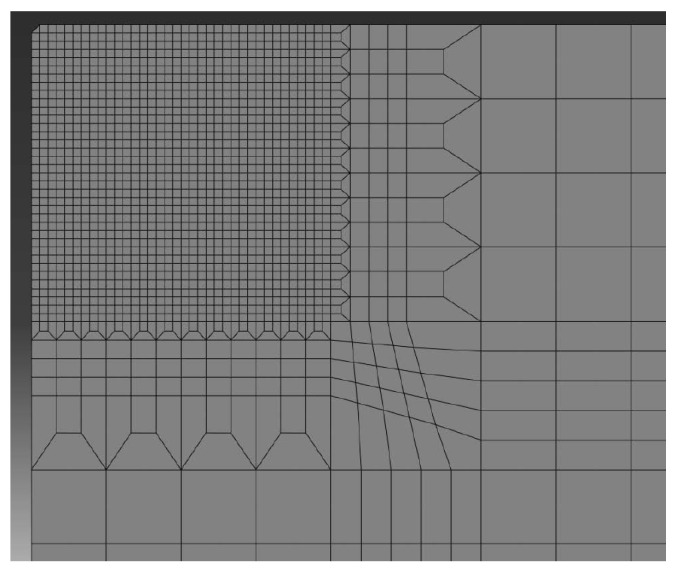
Mesh used; 90,000 elements with a minimum size 0.5 µm.

The example is solved as a 2D symmetric problem in the area the expected crack growth XFEM elements are used (5000). For the energetic description of the material behaviour, the traction separation law obtained by the calibration procedure on 3PB bodies, as described in the previous sections, in Table 1 and Figure 7, is used. The total number of elements exceeded 90,000, but similar results were practically obtained on a mesh for 45,000 elements, see Figure 9.

**Figure 10:**
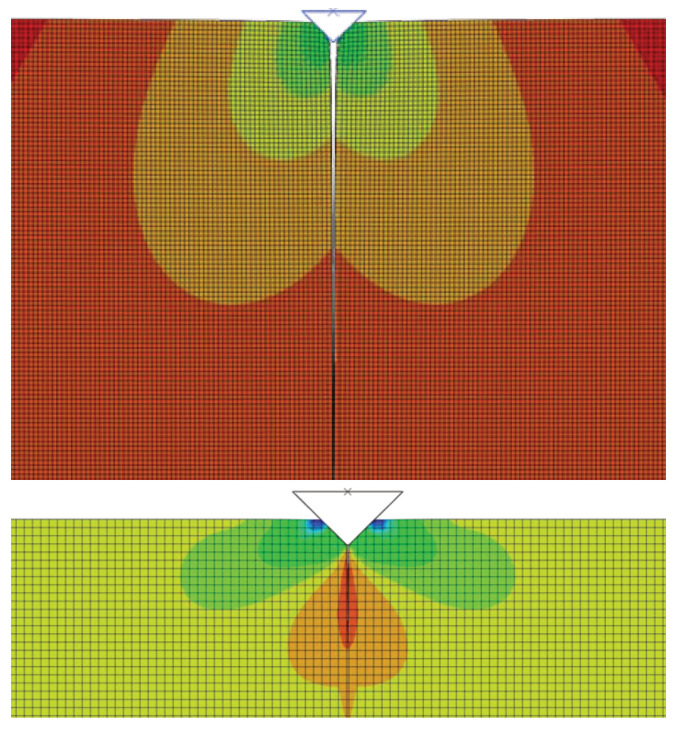
Displacement $u_{1}$ and stress $\sigma_{11}$ distribution for a crack length of 20 µm.

**Figure 11:**
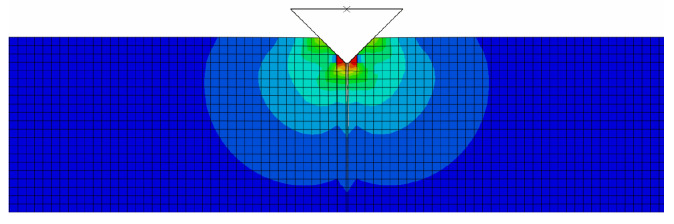
Equivalent Mises stress. The maximum value is about $7.10^{3}$ MPa, and the crack length is about 10 µm.

**Figure 12:**
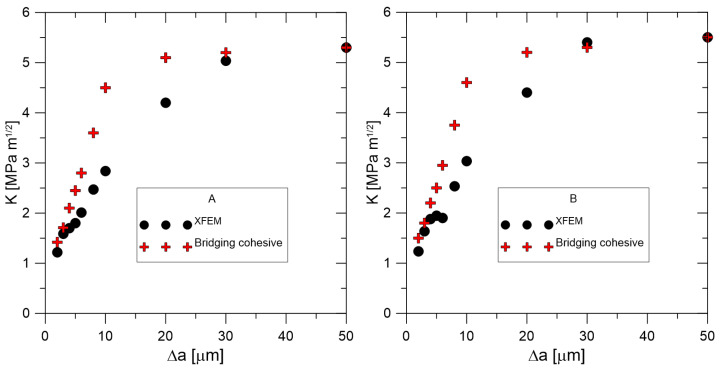
XFEM *J*-*R* curve prediction for the standard traction separation law and for the bridging cohesive model, material A and B.

A detailed description of the crack propagation process may be found in Figure 10 and Figure 11. The indenter is pushed into the matrix and the crack opens, which describes the displacement and stress in the opening directions. To obtain the value of the *J* integral, it was necessary to create a post-processing procedure in MATLAB 24.2 that allows working with incomplete elements. The following Figure 12 shows the calculation results represented by the *J*-*R* curves for the material configurations A and B. At the same time, there are regions for the standard bilinear traction separation law and regions that include the bridging effect.

Using cohesive zone modelling, crack nucleation and propagation for short cracks in the range of 10–100 µm were performed. Fracture behaviour was successfully modelled for different silicon nitride grades by optimising the traction separation parameters. Additionally, the XFEM was successfully applied and provided good results when the bridging effects were not strong (i.e., for longer cracks).

A precise determination of the separation curve’s shape yields a *J*-*R* prediction; at the very least, meticulous experimental procedures are required to determine the maximum stress $\sigma_{0}$. Numerical oscillation is most likely the cause of early real bridging, as evidenced by the smaller displacement values obtained after crack initiation. The ability to forecast or model the behaviour of fibre composites has become crucial since their introduction into technical practice. In addition to solving novel or altered processes, including the existence of solutions, numerical approaches also require that the modelling outcome be distinctly closer to reality. The issue is that a large portion of the input data are estimated, which raises the possibility of an incorrect prediction.

Approaches based on micro scales can be broadly categorised, as in our example, by the multiscale approach, based on the Representative Volume Element (RVE). An RVE is a typical sample of a heterogeneous material that is both significantly smaller than the size of the macroscopic structure and large enough to include enough microheterogeneities to be representative.

## 5. Modifications of the Finite Element and Similar Techniques and Promising Generalizations

**Figure 13:**
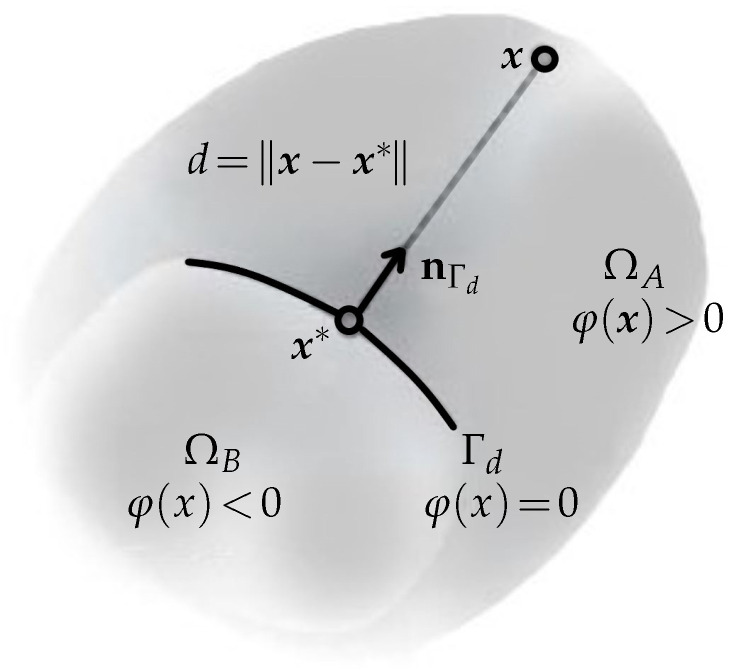
Enrichment function realization: $\varphi \left(x\right)=∥x-x^{*}\;∥\;\mathrm{sgn}\;\left(n_{\Gamma_{d}}\;\cdot \;\left(x-x^{*}\;\right)\right)$ is used for the local creation of the Heaviside function, where $x^{*}$ is the closest point projection of x onto the discontinuity $\Gamma_{d}$, $n_{\Gamma_{d}}\;$ refers to the normal vector to the interface at point $x^{*}$, and $∥x-x^{*}\;∥$ specifies the distance *d* of point x to the discontinuity $\Gamma_{d}$.

Improving the FE approximation basis is one way to enhance the FEM’s performance while using piecewise polynomials to represent singularities, high gradients, or difficulties with oscillatory solutions. It is demonstrated that when piecewise functions are used without enrichment functions, the FE approximation’s rate of convergence is significantly slower than when the approximation basis is enhanced with them. However, the insertion of enrichment functions to approximate the displacement field near the singularity may damage the band structure of the stiffness matrix and lead to an ill-conditioned system. The following two primary needs are met by the enrichment functions that are added to the standard finite element approximation in the XFEM formulation: first, they provide instruction about where the interface should be located within the function space, and second, they provide the possibility to incorporate details regarding the solution’s known asymptotic behaviour nearer to the interface. Therefore, the kind of solution behaviour expected close to the interface specifies the ideal enrichment function for a specific application; see a simple example in Figure 13.

The further development of the XFEM and the related methods is stimulated by the progress in both (i) theory and practice of numerical approaches and (ii) throughput of computers and their systems. Although a robust, reliable, and effective approach for a large class of damage problems is still missing, some advanced approaches for special problems could be inspiring, even for the case of fibre-reinforced composites. Namely, the XFEM enrichment strategies, including some practical convergence considerations, are developed in [49] for hydraulic fractures, e.g., brittle fractures, whose propagation in pre-stressed solid media is induced by the injection of a viscous fluid. The improved XFEM (IXFEM), coming from the comparison of the original XFEM and its intrinsic modification by [38], introduced by [50], tries (i) to suppress some ill-conditioning issues of both these approaches, (ii) to avoid additional DOFs in crack tip enrichments to support the optimization of mass lumping in dynamic calculations, and (iii) to improve contact boundary treatments at all interfaces. A modification of the XFEM for both 2D and 3D problems, is presented in [51,52]. Using global enrichment together with pointwise and integral matching of variables introduced on standard and enriched elements, it aims to derive (if possible) well-conditioned, stable, and optimally convergent algorithms. Ref. [53] demonstrates the computational combination of diffuse damage with discrete crack, with potential applications to quasi-brittle materials. Some mathematical convergence considerations for the XFEM can be found in [54]. The upgrade of the XFEM in [55] combines the approach of [50] with the level set templated cover cutting method (LSTCCM) of [56]. The regularized XFEM (RXFEM) was proposed by [57] to admit arbitrary intersecting 3D cracks, owing to a novel hierarchical enrichment algorithm. Another approach in Ref. [58] combines the XFEM with the multiscale FEM (MsFEM) framework for the analysis of debonding in fibre-reinforced composites.

The formal mathematical theory of the existence of some reasonable (variational, weak, etc.) solutions in infinite-dimensional function spaces and the convergence of sequences of approximate solutions in finite-dimensional ones still contain a lot of open questions and research challenges. However, this is nothing surprising for anybody acquainted with the history of the FEM. Its proper mathematical analysis for 2D elliptic model problems dates back to [59,60] (regular families of triangulation) and [61,62] (quasi-regular families of triangulation), whereas successful computations for significant engineering applications are at least 15 years older, as documented by [63] (published with a certain understandable delay).

The quasi-static approach of [64] for a viscoelastic body, supporting the combination of micro- and macroscopic fracture, cf. [53], avoids the deeper analysis of the quality of the convergence of the XFEM (or a similar method), decomposing an original parabolic problem to a sequence of elliptic problems (still in an infinite-dimensional Sobolev space), using the method of discretisation in time, motivated by the backward Euler method (and its semi-implicit modification), and the convergence properties of Rothe sequences in appropriate Bochner–Sobolev spaces. The approach of [65,66] shows the possibility of extending such results to fully dynamic computations, where an originally problem must be considered as hyperbolic. Unlike these analyses, the approach of [67] goes seemingly back to the application of the GFEM and a relatively simple 2D Poisson problem, but its stable GFEM (SGFEM) guarantees the optimal order of convergence, whereas its conditioning is comparable with that of the standard FEM. The solution of the 2D problem in [68] shows an a priori error estimate for the usual XFEM with a fixed enrichment region as a basis for optimising the XFEM calculations. However, such considerations are limited by a priori knowledge of the allowed crack locations and propagation directions, which simplifies most mathematical proofs but may be inconsistent with the actual physical processes for which a reliable engineering computational interpretation is required. Unfortunately, all attempts to remove such formal mathematical assumptions lead to the much more complicated formulations, not covered by [4] (where the Lipschitz continuous boundary is needed) for Sobolev spaces with generally cracked domains, like [69]. Some useful results, at least for a class of model elliptic interface problems, can be found in the recent study [70], based on the combination of the XFEM with the virtual element method (VEM), as introduced in [71,72].

More details on domains containing cracks—the most convergence results for the XFEM and comparable approaches, including the identification of corresponding limits of sequences of approximate solutions with weak solutions of original initial and boundary value problems for partial differential equations in some reasonable sense—have been (i) derived for simple geometrical configurations, typical for simulations of laboratory experiments, where both the location and the direction for the propagation of a single crack (see, e.g., Figure 10 and Figure 11) or a small number of expectable cracks can be guessed a priori, (ii) or suggested for as an experimental a posteriori error check, with possible controlled remeshing. Unfortunately, the proper mathematical theory for more complicated configurations cannot make use of the existence and convergence results for quasilinear problems from [4], formulated for Lipschitz continuous domains and extensible only to domains composed of a finite number of Lipschitz continuous domains, or domains satisfying the so-called cone property, without serious difficulties. For domains with propagating crack systems, the results of [73,74], together with [69] should be extended and implemented properly, which would require a separate study, beyond the scope of this article.

## 6. Conclusions

Cohesive zone models can be utilised to explore how various material properties, particularly fracture toughness, interact with the local microstructure. The material curve, which reflects the material microstructure, determines the shape of the *J*-*R* curve more than the traction separation law. The crack growth modelling for fibre composites has also been tested via the modified XFEM, and knowledge and practical experience with the usage of a cohesive approach for ductile and brittle fractures have been acquired. The notions of classical fracture mechanics have been applied to the modelling of the process of fracture.

Researchers have focused more on interface issues, in recent years, and have put forward a number of numerical techniques to resolve these issues. One of the most popular techniques for resolving interface issues is the finite element method (FEM). The FEM can be divided into two major types as follows: the interface-fitted FEM and interface-unfitted FEM, depending on whether the mesh fits the interface or not. The interface-unfitted FEM has grown in popularity because creating a high-quality interface-fitted mesh is difficult and time-consuming, especially when the interface changes over time, or the problem includes complex geometry. T. Belytschko et al. initially proposed the extended finite element method (XFEM); this an interface-unfitted FEM and is still under development.

The extended finite element method (XFEM), a powerful tool for structural mechanics, facilitates the development of mechanically enhanced buildings by giving engineers and designers a thorough understanding of how a material structure reacts to stresses. The XFEM approach shows the extraordinary links between material topology and fracture behaviour in engineered materials by improving unexpected fracture toughening mechanisms such as crack deflection and branching. Despite its widespread use, case studies involving XFEM require a thorough revision that emphasises applications, rather than the numerical modelling technique.

The results of the numerical modelling and simulation of the response of deformable bodies and media to external loads with emphasis on their damage behaviour, based on the XFEM and its recent modifications, including the analysis of fibre-reinforced composites, show the advantage of such an approach. The methods are connected with the possibility of advanced adaptive choices for the basis functions, reflecting real physical processes, active on existing and new interfaces. However, the formal mathematical analysis of the convergence of such sequences of approximate solutions still leaves some open questions that are not covered by the lemmas and theorems from the standard functional and numerical analysis. Moreover, such analysis is also influenced by the fact that most computational approaches work with a certain set of both geometrical and physical simplifications, motivated by the selected micromechanical considerations, whereas complete data for suitable scale bridging models, introduced by [23], are not available.

With a focus on highlighting the most recent XFEM research, the application of the XFEM in material design has been shown and discussed, and a quick summary of its applications in other areas has been mentioned. The review can continue with a discussion of the limitations of the XFEM and a summary of its potential applications in applied material science research, including the combination of the XFEM and machine learning methods.

Three categories can then be used to group the latest techniques and algorithms for composites’ progressive damage as follows: (i) trying to produce directly verifiable results, (ii) material input parameters for precise finite element simulations, and (iii) quantifying uncertainty. This discussion elaborates on the existing limitations, difficulties, and future advancements pertaining to machine learning for the progressive damage of composites. It must be acknowledge that to achieve solid agreements between the experiment and modelling results, this often depends more on understanding the physical processes in the given material. Improvements in the numerical methods can be seen in the application of the XFEM to solving dynamic problems in the area of very fast processes.

## Figures and Tables

**Table 1:** Data for the determined bridging law.

	$J_{0}$	$\Delta J_{\mathrm{SS}}$	$\Delta u_{2}$	$\Delta u_{c}$	$\Delta u_{1}$	∑J	∑K
Material	[J/m^2^]	[J/m^2^]	[mm]	[mm]	[mm]	[J/m^2^]	[MPa m^−1/2^]
A	16.4	29.6	0.0125	0.15	0.050	46.0	3.82
B	8.6	38.2	0.0125	0.25	0.075	46.8	3.86
C	20.2	58.2	0.0200	0.30	0.082	78.4	4.99

## Data Availability

The data presented in this study are available upon request from the corresponding author.

## References

[1] Papenfuß C. (2020). Continuum Thermodynamics and Constitutive Theory.

[2] Fedele R., Placidi L., Fabbrocino F. (2024). A review of inverse problems for generalized elastic media: Formulations, experiments, synthesis. Continuum Mech. Thermodyn..

[3] Sumi I. (2014). Mathematical and Computational Analyses of Cracking Formation.

[4] Roubíček T. (2005). Nonlinear Partial Differential Equations with Applications.

[5] Khoei A.R. (2014). Extended Finite Element Method: Theory and Applications.

[6] Xiao G., Wen L., Tian R. (2021). Arbitrary 3D crack propagation with Improved XFEM: Accurate and efficient crack geometries. Comput. Methods Appl. Mech. Eng..

[7] Song J.-H.H., Areias P.M.A., Belytschko T. (2006). A method for dynamic crack and shear band propagation with phantom nodes. Int. J. Numer. Methods Eng..

[8] Wen L., Tian R. (2016). Improved XFEM: Accurate and robust dynamic crack growth simulation. Comput. Methods Appl. Mech. Eng..

[9] Kozák V., Vala J. (2024). Use of cohesive approaches for modelling critical states in fibre-reinforced structural materials. Materials.

[10] Ma J., Chen W., Zhang C., Lin J. (2020). Meshless simulation of anti-plane crack problems by the method of fundamental solutions using the crack Green’s function. Comput. Math. Appl..

[11] Zhang M., Abidin A.R.Z., Tan C.S. (2024). State-of-the-art review on meshless methods in the application of crack problems. Theor. Appl. Fract. Mech..

[12] Lendauer M., Danzer R. (2008). Silicon nitride tools for the hot rolling of high-alloyed steel and superalloy wires—Crack growth and lifetime prediction. J. Eur. Ceram. Soc..

[13] Lange F.F. (1979). Fracture toughness of Si_3_N_4_ as a function of the initial *α*-phase content. J. Am. Ceram. Soc..

[14] Hoffmann M.J., Petzow: G. (1994). Tailored microstructures of silicon nitride ceramics. Pure Appl. Chem..

[15] Supancic P., Danzer R., Witschnig S., Polaczek E., Morrell R. (2009). A new test to determine the tensile strength of brittle balls—The notched ball test. J. Euro. Ceram. Soc..

[16] Fünfschilling S.F., Fett T., Hoffmann M.J., Oberacker R., Schwind T., Wippler J., Böhlke T., Özcoban H., Schneider G.A., Becher P.F. (2011). Mechanisms of toughening in silicon nitrides: The roles of crack bridging and microstructure. Acta Mater..

[17] Greene R.B., Fünfschilling S.F., Fett T., Hoffmann M.J., Ager J.W., Kruzic J.J. (2014). Fatigue threshold *R*-curves predict fatigue endurance strength for self-reinforced silicon nitride. J. Am. Ceram. Soc..

[18] Cotterel B., Atkins A.G. (1996). A review of the *J* and *I* integrals and their implications for crack growth resistance and toughness in ductile fracture. Int. J. Fract..

[19] Meindlhumer M., Alfreider M., Sheshi N., Hohenwarter A., Todt J., Rosenthal M., Burghammer M., Salvati E., Keckes J., Kiener D. (2025). Resolving the fundamentals of the *J*-integral concept by multi-method in situ nanoscale stress–strain mapping. Commun. Mater..

[20] Kozák V., Chlup Z., Padělek P., Dlouhý I. (2017). Prediction of traction separation law of ceramics using iterative finite element method. Solid State Phenom..

[21] Govindasamy L., Park Y.-J., Ko J.-W., Lee J.-W., Ma H.-J., Kumar K., Kim H.-N. (2024). Role of *β*-Si_3_N_4_ seeds in microstructure development and properties of silicon nitride ceramics: A comprehensive review. J. Korean Ceram. Soc..

[22] Zhang Q., Wang W., Zhang Z., Liang Y., Sun F., Wang Z., Han G., Zhang W. (2024). Enhancing fracture toughness of silicon nitride ceramics by addition of *β*-Si_3_N_4_ whisker and MXene. Ceram. Int..

[23] Steinhauser M.O. (2008). Computational Multiscale Modelling of Fluids and Solids.

[24] Xie J., Zhing N. (2024). Microscale and mesoscale modeling for compressive failure in unidirectional carbon fiber reinforced polymer composites with random distribution of fiber misalignments. Polym. Compos..

[25] Tang Y.-Q., Yin Z.-Y., Jin Y.-F., Zhou X.-W. (2024). A novel mesoscale modelling method for steel fibre-reinforced concrete with the combined finite-discrete element method. Cem. Concr. Compos..

[26] Belytschko T., Gracie R., Ventura G. (2009). A review of extended/generalized finite element methods for material modeling. Modell. Simul. Mater. Sci. Eng..

[27] Zi G., Belytchko T. (2003). New crack-tip elements for XFEM and applications to cohesive cracks. Int. J. Numer. Meth. Eng..

[28] Deng H., Yan B., Zhang X., Zhu Y., Koyanagi J. (2023). New crack front enrichment for XFEM modeling. Int. J. Solids Struct..

[29] Abdelaziz Y., Hamouine A. (2008). A survey of the extended finite element. Comput. Struct..

[30] Cervera M., Barbat G.B., Chiumenti M., Wu J.Y. (2022). A comparative review of XFEM, mixed FEM and phase-field models for quasi-brittle cracking. Arch. Comput. Methods Eng..

[31] Vellwock A.E., Libonati F. (2024). FEM for composites, biological, and bioinspired materials: A review. Materials.

[32] Swati R.F., Wen L.H., Elahi H., Khan A.A., Shad S. (2019). Extended finite element method (XFEM) analysis of fiber reinforced composites for prediction of micro-crack propagation and delaminations in progressive damage: A review. Microsyst. Technol..

[33] Huang S., Fu Q., Yan L., Kasal B. (2021). Characterization of interfacial properties between fibre and polymer matrix in composite materials—A critical review. J. Mater. Res. Technol..

[34] Loh J.Y.Y., Yeoh K.M., Raju K., Pham V.N.H., Tan V.B.C., Tay T.E. (2024). A review of machine learning for progressive damage modelling of fiber-reinforced composites. Appl. Compos. Mater..

[35] Melenk J.M., Babuška I. (1996). The partition of unity finite element method: Basic theory and applications. Comput. Methods Appl. Mech. Eng..

[36] Babuška I., Strouboulis T., Copps K. (2000). The design and analysis of the generalized finite element method. Comput. Methods Appl. Mech. Eng..

[37] Moës N., Dolbow J., Belytschko T. (1999). A finite element method for crack growth without remeshing. Int. J. Numer. Methods Eng..

[38] Fries T.-P., Belytchko T. (2006). The intrinsic XFEM: A method for arbitrary discontinuities without additional unknowns. Int. J. Numer. Methods Eng..

[39] Simone A. (2007). Partition of unity-based discontinuous finite elements: GFEM, PUFEM, XFEM. Rev. Eur. Genie Civ..

[40] Wang Y., Javadi A.A., Fidelibus C., Liang H. (2024). Improvements for the solution of crack evolution using extended finite element method. Nat. Sci. Rep..

[41] Needleman A. (1992). Micromechanical modelling of interfacial decohesion. Ultramicroscopy.

[42] Pike M.G., Oskay C. (2005). XFEM modelling of short microfibre reinforced composites with cohesive interfaces. Finite Elem. Anal. Des..

[43] Kaliske M. (2000). A formulation of elasticity and viscoelasticity for fibre reinforced material at small and finite strains. Comput. Methods Appl. Mech. Eng..

[44] Endo V.T., de Carvalho Pereira J.C. (2017). Linear orthotropic viscoelasticity model for fiber reinforced thermoplastic material based on Prony series. Mech. Time-Depend. Mater..

[45] Guangwu F., Long L., Xiguang G., Yingdong S. (2020). Finite element analysis of the crack deflection in fiber reinforced ceramic matrix composites with multilayer interphase using virtual crack closure technique. Appl. Compos. Mater..

[46] Nguyen-Thanh N., Li W., Huang J., Zhou K. (2022). Multi phase-field modeling of anisotropic crack propagation in 3D fiber-reinforced composites based on an adaptive isogeometric meshfree collocation method. Comput. Methods Appl. Mech. Eng..

[47] Kozák V., Vala J. (2023). Crack growth modelling in cementitious composites using XFEM. Procedia Struct. Integr..

[48] Xu W.-J., Shi X., Sun Y., Feng S.-Q., Zhao Y.-L. (2024). Fracture mechanics model of biological composites reinforced by helical fibers. Compos. Struct..

[49] Gordeliy E., Peirce A. (2015). Enrichment strategies and convergence properties of the XFEM for hydraulic fracture problems. Comput. Methods Appl. Mech. Eng..

[50] Tian R., Wen L. (2015). Improved XFEM—An extra-dof free, well-conditioning, and interpolating XFEM. Comput. Methods Appl. Mech. Eng..

[51] Agathos K., Chatzi E., Bordas S.P.A., Talaslidis D. (2016). A well-conditioned and optimally convergent XFEM for 3D linear elastic fracture. Int. J. Numer. Meth. Eng..

[52] Agathos K., Ventura G., Chatzi E., Bordas S.P.A. (2018). Stable 3D XFEM/vector level sets for non-planar 3D crack propagation and comparison of enrichment schemes. Int. J. Numer. Meth. Eng..

[53] Lu X., Guo X.M., Tan V.B.C., Tay T.E. (2021). From diffuse damage to discrete crack: A coupled failure model for multi-stage progressive damage of composites. Comput. Methods Appl. Mech. Eng..

[54] Liu G., Guo J., Bao Y. (2022). Convergence investigation of XFEM enrichment schemes for modeling cohesive cracks. Mathematics.

[55] Wang L.-X., Wen L.-F., Tian R., Feng C. (2024). Improved XFEM (IXFEM): Arbitrary multiple crack initiation, propagation and interaction analysis. Comput. Methods Appl. Mech. Eng..

[56] Wen L.-F., Tian R., Wang L.-X., Feng C. (2023). Improved XFEM for multiple crack analysis: Accurate and efficient implementations for stress intensity factors. Comput. Methods Appl. Mech. Eng..

[57] Iarve E.V., Zhou E., Ballard K.M., Gao Z., Adluru H.K., Mollenhauer D. (2025). Regularized X-FEM modeling of arbitrary 3D interacting crack networks. Int. J. Numer. Methods Eng..

[58] Liu G., Guo J., Bao Y., Zhu H., Sun K. (2024). Multiscale simulation of debonding in fiber-reinforced composites by a combination of MsFEM and XFEM. Eng. Fract. Mech..

[59] Aubin J.P. (1967). Approximation des espaces de distributions et des opérateurs différentiels. Mém. Soc. Math. Fr..

[60] Zlámal M. (1968). On the finite element method. Num. Math..

[61] Jamet P. (1976). Estimations d’erreur pour des éléments finis droits presque dégénérés. RAIRO Anal. Numér..

[62] Křížek M. (1991). On semiregular families of triangulations and linear interpolation. Appl. Math..

[63] Turner M.J., Clough R.W., Matrin H.C., Topp L.J. (1956). Stiffness and deflection analysis of complex structures. J. Aeronaut. Sci..

[64] Vala J., Kozák V. (2020). Computational analysis of quasi-brittle fracture in fibre reinforced cementitious composites. Theor. Appl. Fract. Mech..

[65] Vala J., Kozák V. (2021). Non-local damage modelling of quasi-brittle composites. Appl. Math..

[66] Vala J. (2023). Numerical approaches to the modelling of quasi-brittle crack propagation. Arch. Math..

[67] Zhang Q., Babuška I., Banerjee U. (2016). Robustness in stable generalized finite element methods (SGFEM) applied to Poisson problems with crack singularities. Comput. Methods Appl. Mech. Eng..

[68] Nicaise S., Renard Y., Chahine E. (2011). Optimal convergence analysis for the extended finite element method. Int. J. Numer. Meth. Eng..

[69] Chandler-Wilde S.N., Hewett D.P., Moiola A. (2017). Sobolev spaces on non-Lipschitz subsets of *R*^n^ with application to boundary integral equations on fractal screens. Integr. Equ. Oper. Theory.

[70] Zheng X., Chen J., Wang F. (2024). An extended virtual element method for elliptic interface problems. Comput. Math. Appl..

[71] Mascotto L. (2023). The role of stabilization in the virtual element method: A survey. Comput. Math. Appl..

[72] Chen Y., Sun D., Perego U., Li Q. (2025). Brittle crack propagation simulation based on the virtual element method and *J*_k_-integral fracture criterion. Eng. Fract. Mech..

[73] Burenkov V.I. (1998). Sobolev Spaces on Domains.

[74] Cianchi A., Maz’ya V.G. (2016). Sobolev inequalities in arbitrary domains. Adv. Math..

